# *Helicobacter pylori *and gastroduodenal pathology: New threats of the old friend

**DOI:** 10.1186/1476-0711-4-1

**Published:** 2005-01-05

**Authors:** Niyaz Ahmed, Leonardo A Sechi

**Affiliations:** 1 Pathogen Evolution Group, Centre for DNA Fingerprinting and Diagnostics (CDFD), Hyderabad, India; 2 Department of Biomedical Sciences, University of Sassari, Sassari, Italy

## Abstract

The human gastric pathogen *Helicobacter pylori *causes chronic gastritis, peptic ulcer disease, gastric carcinoma, and mucosa-associated lymphoid tissue (MALT) lymphoma. It infects over 50% of the worlds' population, however, only a small subset of infected people experience *H. pylori*-associated illnesses. Associations with disease-specific factors remain enigmatic years after the genome sequences were deciphered. Infection with strains of *Helicobacter pylori *that carry the cytotoxin-associated antigen A (*cagA*) gene is associated with gastric carcinoma. Recent studies revealed mechanisms through which the cagA protein triggers oncopathogenic activities. Other candidate genes such as some members of the so-called plasticity region cluster are also implicated to be associated with carcinoma of stomach. Study of the evolution of polymorphisms and sequence variation in *H. pylori *populations on a global basis has provided a window into the history of human population migration and co-evolution of this pathogen with its host. Possible symbiotic relationships were debated since the discovery of this pathogen. The debate has been further intensified as some studies have posed the possibility that *H. pylori *infection may be beneficial in some humans. This assumption is based on increased incidence of gastro-oesophageal reflux disease (GERD), Barrett's oesophagus and adenocarcinoma of the oesophagus following *H. pylori *eradication in some countries. The contribution of comparative genomics to our understanding of the genome organisation and diversity of *H. pylori *and its pathophysiological importance to human healthcare is exemplified in this review.

## Introduction

*Helicobacter pylori *is a bacterium that colonizes the harshly acidic milieu of the human stomach. More than half of the world's population carries this infection. Infection rates vary among the developed and developing countries of the world. *H. pylori *infection is on a steep decline in most of the western countries mainly due to the success of combination therapies and improved personal hygiene and community sanitation to prevent re-infection. However, the situation is not improving in many of the developing countries due to failure of treatment regimes and emergence of drug resistance. The infection in some cases leads to chronic superficial gastritis, chronic active gastritis, peptic ulcer disease and gastric adenocarcinoma [1-4]. One of the most distinctive features of *H. pylori *is the genetic diversity between clinical isolates obtained from different patient populations. Most *H. pylori *isolates can be discriminated from others by DNA profiling [5-8] or sequencing of corresponding genes due to mainly a high degree of sequence divergence between orthologs (3–5%) [9,10]. Also, *H. pylori *has a panmictic or freely recombining population structure [11] and is naturally competent [12]. These characteristics facilitate an inter-strain recombination due mainly to horizontal exchange of alleles from other strains colonising the same niche, which is extremely common in *H. pylori *chromosome. However, such genetic recombinations in the *H. pylori *genome might not be deleterious because they occur in the plasticity zone, a special cluster of DNA rearrangements that protects the essential complement of genes by acting as a bed for foreign DNA insertion or abrogation. DNA loss and rearrangement are therefore a norm for *H. pylori*, and flexibility and diversity in gene content may contribute to bacterial fitness in different members of the diverse human host population. Post genomic analyses have revealed interesting attributes of *H. pylori *pathogenicity and novel mechanisms of causation of ulcer disease and cancer have been envisaged. Efforts to know the cause and potential benefits of the genetic diversity of this bacterium has led to some interesting discoveries relating to its co-evolution with the human host, microevolution while during infection and quasi-species development, virulence determinants and eradication strategies. Recent studies reviewed herein collectively aim at testing the speculation about whether *H. pylori *may be beneficial to human health in certain circumstances and whether eradication of this organism is always necessary? Epidemiological studies are needed in the context of such intriguing hypotheses. Results obtained from such studies might enable the development of a high-throughput screening system for high-risk groups within the huge population of *H. pylori*-infected individuals. Recent studies have shown that *H. pylori *infection protects against gastro-oesophageal reflux and oesophageal carcinoma. So it will be important to selectively eradicate *H. pylori *in people that are at the highest risk of developing gastric carcinoma. Eradication of *highly pathogenic H. pylori *specifically from high-risk groups would markedly reduce the worldwide incidence of ulcer and gastric cancer.

### Epidemiology and Evolution

*H. pylori *infection is usually acquired during childhood, where transmission occurs predominantly within families [13]. A couple of recent studies demonstrated the possible co-existence of a large array of clonal lineages within *H. pylori *populations that are evolving in each individual separately from one another [14,10]. It is therefore probable that via this semi-vertical transmission of *H. pylori *strains, there are distinct sets of *H. pylori *genotypes colonising different human populations. With different strains evolving separately of one another and the fact that *H. pylori *is a genetically diverse (panmictic) organism, distinct genotypes have been found to be associated with particular geographic regions [15,16]. For example, the shuffling of variant regions within the *vacA *gene (a gene encoding a vacuolating cytotoxin) within a local *H. pylori *population has led to predominant *vacA *genotypes being characteristic of isolates from different geographic regions. In addition, worldwide studies encompassing *H. pylori *isolates from many geographic regions have demonstrated weak clonal groupings and geographic partitioning of *H. pylori *isolates [9,17]. If recombination only occurs between a resident *H. pylori *population, exchange of genetic sequences can genetically homogenise this population. As *H. pylori *is naturally competent and recombination occurs frequently [11], specific genotypes associated with different geographic regions occur as a result of this homogenising force.

Introduction of polymorphisms and sequence variants from one *H. pylori *population from a particular geographic region to another *H. pylori *population from another geographic region *via *human migration makes the association of particular genotypes with specific geographic locations more difficult. Although the introduction of new polymorphisms into a particular *H. pylori *population poses a problem with identifying specific genotypes within certain geographic locales, it may, however, provide information on the ancestry of the hosts in whose stomachs the strains were carried. Studies have been aimed at demonstrating the path of human migration to Latin America with conflicting results regarding whether European or Asian populations brought *H. pylori *to South America [16,11]. However, a recent and comprehensive study by Flaush *et al*. [19] demonstrated that sequence analysis of *H. pylori *isolates recovered from twenty-seven countries displayed geographic partitioning. Thus, polymorphisms within the *H. pylori *genome can serve as useful markers for studying ancient human migrations. However, a mix up of *H. pylori *strains between migrated and native populations can sometimes complicate analysis. Accordingly, the study of migrated populations that have remained isolated from the native populations is essential.

#### Genome organization, genetic diversity and microevolution

Since its successful isolation in 1983 by Warren and Marshall, *H. pylori *has been linked to various pathologies and a strong association with gastric carcinoma and mucosa-associated lymphoid tissue lymphoma [1,3] has been established. However, although *H. pylori *is definitely responsible for these diseases, only less than 10% of people colonized with *H. pylori *portray disease symptoms. This suggests that specific *H. pylori *strains may be responsible for virulence in different hosts. Many studies have shown that certain allotypes of the *vacA *gene and the presence a functional *cagA *gene are associated with an increased risk of peptic ulceration and gastric cancer, respectively [20-22]. However, these correlations vary based on the host population studied and efforts to correlate other *H. pylori *alleles with clinical diseases have failed.

So how do a few *H. pylori *strains trigger higher virulence as compared to other strains? Current approaches in functional genomics based on protein- protein interaction and microarray based transcription profiling are helping to decode this mystery. Functional genomics often uses the gene chip based expression profiling to provide a condition-dependent and time-specific genome wide profile of an organism's transcriptome [23,24]. Whereas, comparative genomics juxtaposes two or more genome sequences at the level of gene content and organization [25,26]. Both the approaches harness extensive computer algorithms and *in silico *modelling to summarise gene encoded (or putative) functions. *H. pylori *has been the first prokaryote wherein full genome sequence of two different patient isolates [J99 and 26995] were characterized and compared [27,28]. As *H. pylori *is a freely recombining or panmictic organism [11] the question of whether the two genome sequences would accurately represent the myriad of genetic diversity found among the strains was posed. Since the 2 sequenced strains were obtained a decade apart from the two different continents and cultured from the lesions of different gastric disorders, it has been widely assumed that their genome sequences will more likely portray the genetic diversity exhibited by clinical isolates. Comparative genomic analysis of the two completely sequenced strains revealed a significant amount of genetic variation between their genomes. For instance, the J99 genome is shorter (1,643,831 bp) than that of strain 26695 (1,667,867 bp) and has 57 less predicted ORF's [27,28]. Strains 26695 and J99 contain 110 and 52 strain specific genes, respectively [29,30], in which more than a half reside within a locus termed the plasticity cluster. A recent approach helped revised annotation and comparison of the two sequenced *H. pylori *genomes [31] and reclassified the coding sequences. Based on this study the total number of hypothetical proteins was reduced from 40% to 33%. A large amount of size variation was also discovered between orthologous genes mostly due to natural polymorphisms arising as a result of natural transformation and free recombination within *H. pylori *chromosome. Recombinational events including the presence of insertion elements, pathogenicity islands, horizontally acquired genes (restriction recombination genes), mosaics and chromosomal rearrangements were frequently annotated in subsequent bioinformatics based attempts. It has been argued that such diversity is a result of a lack of direct competition between strains, even when resident within different individuals within the same community [32]. However, a recent study has demonstrated that integration of foreign gene fragments acquired via natural transformation is often prevented by the well-developed restriction-modification systems in *H. pylori *genome [33]. It has been demonstrated that *H. pylori *has extensive, non-randomly distributed repetitive chromosomal sequences, and that recombination between identical repeats contributes to the variation within individual hosts [34]. That *H. pylori *is representative of prokaryotes, especially those with smaller (<2 megabases) genomes, that have similarly extensive direct repeats, suggests that recombination between such direct DNA repeats is a widely conserved mechanism to promote genome diversification [33]. In addition, although *H. pylori *has been termed as a panmictic organism [11,35], it is surprising that clonal lineages within *H. pylori *populations exist [36-39]. Recent reports demonstrated that *H. pylori *in some populations shows a clonal descent and suggest that a large array of *H. pylori *clonal lineages co-exist, which evolve in isolation from on another [14]. Moreover, in certain parts of the world *H. pylori *isolates have been shown to exhibit little genetic heterogeneity, based on fingerprint profiles [17,40]. Functional genomics, utilising microarray technology, has provided researchers with a powerful tool to investigate the genetic diversity of clinical isolates [41], the transcriptional profiles of isolates grown under different conditions [42], the identification of strain-specific and species-specific genes [29,30] and the diversity between strains giving rise to differing clinical illnesses [43]. One of the interesting findings using microarray based genotyping has been the discovery that *H. pylori *isolates undergo 'microevolution' and give rise to sub-species during prolonged colonisation of a single host [44-46]. The presence of stable sub-species within a single individual suggests an adaptation of a *H. pylori *population to specific host niches, facilitated by unknown advantages conferred to them by select plasticity region genes. Bjorkholm *et al*. [45] demonstrated that several loci differed within two genetically related isolates from the same host, one major difference being the presence of the *cag *pathogenicity island (*cag *PAI) in one isolate and not the other. As the *cagA *gene and *cag *PAI are principle virulence factors within the strains, the excision or abrogation of the *cag *PAI within a strain may indicate that attenuating the virulence of a strain could be a favourable adaptation.

#### The conundrum of strain diversity: How many more genome sequences do we need to understand this bug?

Within the bacterial populations, genome content may not be fixed, as changing selective forces favour particular phenotypes; however, organisms well adapted to particular niches may have evolved mechanisms to facilitate such plasticity. The highly diverse *H. pylori *is a model for studying genome plasticity in the colonization of individual hosts. For *H. pylori*, neither point mutation, nor intergenic recombination requiring the presence of multiple colonizing strains, is sufficient to fully explain the observed diversity.

The two *H. pylori *genomes sequenced to date are each from ethnic Europeans, and genomic comparisons modelled on these data are sufficient to identify novel loci from new strains, especially from understudied Asian populations. However, these genome sequences may not be fully representative of the entire diversity of the gene pool. Identification and characterization of such loci which are more abundant in the Asian gene pool may lead to newer insights into the mechanisms of *H. pylori *colonization, carriage and virulence in the countries of Asia which are more seriously under threat from *H. pylori*. Therefore, future high throughput efforts involving a large number of strains are clearly needed. Taking the Indian example for instance, according to the Ethnologue database , there are about 1683 languages and dialects ('mother tongues') in this country and *H. pylori *diversity therefore can be assumed to coincide with this figure. So one has to roughly look at the inter-strain genomic diversity contributed by approximately 1683 different strains representing each dialect and or a community.

Nonetheless, genotypic data from each geographic area or a community is extremely vital and might constitute a missing piece of a large, biologic jigsaw puzzle.

### Natural competence and transformation

**Figure 1:**
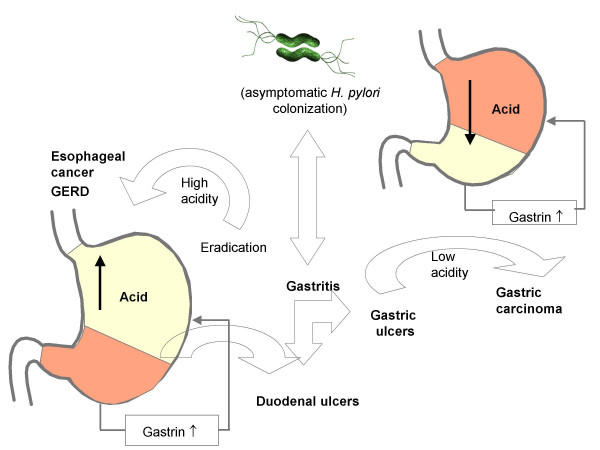
Different outcomes of *H. pylori *infection. Some studies argue that eradication of *H. pylori *might trigger some of the worst forms of heart burns and increased acidity, leading ultimately to oesophageal cancer and or GERD.

Independent of the other two pathogenesis associated type IV systems, *H. pylori *harbours a dedicated type IV apparatus, the comB gene cluster [47] linked to the natural transformation and competence. The *comB *gene cluster is essential for the bacterium to take up plasmid and chromosomal DNA during natural transformation. To identify the genes essential for natural transformation competence in *H. pylori*, genetic approach of transposon shuttle mutagenesis has been used and the *comB *locus was located, consisting of *orf2 *and *comB1-comB3 *[48,49]. This cluster contains four tandemly arranged genes, ORF2, comB1, comB2 and comB3 as a single transcriptional unit. Subsequently, the components of comB cluster namely Orf2, comB1, comB2 and comB3 were renamed (according to homology with the *Agrobacterium tumifaciens *type IV secretory apparatus) as comB7, comB8, comB9 and comB10 respectively (Figure 1). Another ORF in HP26695, HP0017 was found to be homologous to the virB4 gene in *Agrobacterium tumifaciens *type IV secretory apparatus and was named as comB4 [50]. From this study it also appeared that each of the gene products of ORFs comB8 to comB10 were absolutely essential for the development of natural transformation competence. It appears that the comB transformation apparatus has evolved conservatively and is typically present in all the strains. This conservation is interestingly in agreement with the need for genomic fluidity in *H. pylori *where deletions and rearrangements due to natural transformation and transposition are the norm. This is therefore necessary for the pathogen to keep the gene content flexible and as diverse as possible to probably acclimatise itself to diverse host niches during the process of infection. Both these systems, the *cag*-PAI encoded type IV export system and the transformation-associated type IV system seem to act completely independently, since the deletion of one system from the chromosome does apparently not affect the function of the other system.

### Pathogenic apparatuses

#### The cag pathogenicity island

Molecular analysis of bacterial transport has been attempted in several bacterial pathogens. Among such transport systems, Type IV secretion systems have been described in greater detail in diverse bacteria. In *H. pylori*, 3 different kinds of type IV secretion apparatuses have been identified. The first such secretion system identified in *H. pylori *was the one comprised of 29 genes encoding the cag pathogenicity island (cag-PAI). One of the principal virulence factors of *H. pylori*, the cagA antigen is contained in the 40 kb cag-PAI. The tyrosine-phosphorylated cagA protein is translocated to the epithelial cells by the type IV secretion system (forming a sort of syringe like structure) [51-53]. Upon tyrosine phosphorylation, the cagA protein elicits growth factor like stimuli in epithelial cells (hummingbird phenotype) coupled with interleukin-8 induction for the recruitment of neutrophils. Mutations in several genes of the *cag*-PAI interfere with tyrosine phosphorylation and induction of interleukin-8 secretion [54]. In recent studies, in order to analyse which genes of the *cag*-PAI are essential for cagA translocation and/or interleukin 8 induction, a complete mutagenesis of the *cag*-PAI was performed [55]. In general, it appears that most of the cag genes are involved in assembly and arrangement of the secretory apparatus. Five of these genes namely HP0524 (virD4), HP0525 (virB11), HP0527 (virB10), HP0528 (virB9) and HP0544 (virB4/cagE) constitute the main apparatus of the type IV secretory system of *H. pylori *[56]. All these genes except HP0524 are associated with IL8 production [55]. However, the presence of strains eliciting IL 8 responses irrespective of intactness of the cag-PAI underlines the fact that it's 'not' the only factor linked to IL8 secretion [57].

Very recently, and for the first time, ultrastructure analysis of the surface of *H. pylori *26695 has revealed a sheathed, surface organelle, coded by the cag-PAI genes, HP0527 (forms sheath around the pilus needle) and HP0532/ cagT (forms the base of the pilus) [58]. This structure, although uncommon, could be the special adaptation of *H. pylori *to the host niches and this might mediate biological as well as transport functions of the cag-PAI encoded proteins. Computational analyses to predict the macromolecular assemblies of such apparatuses are needed to have a more simplified understanding of the entire model of the *H. pylori *type IV secretion mechanism

#### Link with cancer: the oncogenic cagA protein

A large-scale prospective study revealed that the risk for development of gastric carcinoma was much greater in the *H. pylori*-infected population than in the *H. pylori*-uninfected population [59]. The *cagA *gene of *H. pylori *is assumed as partially responsible for eliciting signaling mechanisms that lead to the development of gastric adenocarcinoma. Based on the carriage of a functional *cagA *as a marker for the *cag *PAI, the *H. pylori *species is divided into *cagA*-positive and *cagA*-negative strains. The *cagA*-positive strains are associated with higher grades of gastric or duodenal ulceration and are more virulent than the *cagA*-negative strains [60]. Some epidemiological studies have demonstrated roles of *cagA *positive *H. pylori *in the development of atrophic gastritis, peptic-ulcer disease and gastric carcinoma [61,62]. The *cagA *gene product, cagA, is translocated to the gastric epithelial cells to undergo tyrosine phosphorylation by SRC family kinases [63]. Tyrosine phosphorylation is known to occur at the EPIYA motifs on the cagA. The cagA protein upon phosphorylation binds and activates a SHP2 phosphatase that acts as a human oncoprotein. As SHP2 transmits positive signals for cell growth and motility, deregulation of SHP2 by cagA is an important mechanism by which *cagA*-positive *H. pylori *promotes gastric carcinogenesis. Cag A is noted for its variation at the SHP2 binding site and, based on the sequence variation, it is sub- classified into two main types – East-Asian cagA and Western cagA. East-Asian cagA shows stronger SHP2 binding and greater biological activity than Western cagA. In East-Asian countries, endemic circulation of *H. pylori *strains that carry biologically active forms of cagA might underlie the high incidence of gastric carcinoma. One puzzling attribute of *H. pylori *infection is why some populations with high incidences of *H. pylori *infection, such as those in Japan and Korea, have high incidences of gastric carcinoma, whereas other highly infected populations, such as populations in central Africa, do not. Possible reasons could be the differences in genetic susceptibility among populations, environmental factors such as dietary habits, and strain differences of *H. pylori*. Among these, diversity of cagA in *H. pylori *strains might be involved in determination of the type and severity of disease. As discussed above, East-Asian and Western forms of cagA possess the distinctly structured tyrosine phosphorylation/ SHP2-binding sites – EPIYA-D and EPIYA-C, respectively [64]. Notably, the grades of inflammation, activity of gastritis, and atrophy are significantly higher in patients with gastritis who were infected with the East-Asian cagA-positive strain than in patients infected with the cagA-negative or Western cagA-positive strain [65]. Furthermore, the prevalence of the East-Asian *cagA*-positive strain is associated with the mortality rate of gastric cancer in Asia. Therefore, populations infected with East-Asian *cagA *positive *H. pylori *are at greater risk for gastric cancer than those infected with Western *cagA*-positive strains.

Among Western CagA species, the number of EPIYA-C sites directly correlates with levels of tyrosine phosphorylation, SHP2-binding activity and morphological transformation [64]. Furthermore, molecular epidemiological studies have shown that the number of EPIYA-C sites is associated with the severity of atrophic gastritis and gastric carcinoma in patients infected with Western CagA-positive strains of *H. pylori *[66].

### The number-2 virulence determinant: vacuolating cytotoxin (*Vac*A) of *H. pylori*

*H. pylori *has a single copy of the *vacA *gene. Screening of *H. pylori *chromosomal fragments permitted the identification of a 3864-base pair open reading frame (*vacA*) that encoded the vacuolating cytotoxin [67]. The sequence of the *vacA *gene includes a 33 amino acid signal sequence. With the exception of a hydrophobic region at the N terminus, the mature 90-kDa protein (amino acids 34 to 842) is mainly hydrophilic [67]. The cytotoxic activity of VacA has been shown to increase substantially under acidic conditions. VacA protein, a secreted 95 kD peptide, varies in the signal sequence (alleles s1a, s1b, s1c, s2) and/or its middle region (alleles m1, m2) between different *H. pylori *strains. The different combinations of s and m regions determine the production of cytotoxic activity. Strains with the genotype s1 m1 produce high levels of vacuolating cytotoxin in vitro. Strains with the genotype s2 produce an inactive toxin. Whereas, strains with the genotype m2 produce toxic activity with a different target cell specificity from those of m1 genotype. Genotypic variations in the vacA gene structure specific to a geographic locale have been recognised. While the vacA m1a allele is specific for the European strains [16], the vacA m1b genotype is typical of the Asian strains [68]. Yet another signal region genotype, s1c is also characteristic of the East Asians [69]. Among other functions, VacA selectively inhibits the invariant chain (Ii)-dependent pathway of antigen presentation mediated by the MHC class II and might induce apoptosis in epithelial cells. VacA, so far mainly regarded as a cytotoxin of the gastric epithelial cell layer, now turns out to be a potent immunomodulatory toxin, targeting the adapted immune system. Thus, in addition to the well-known vacuolating activity, VacA has been reported to induce apoptosis in epithelial cells, to affect B lymphocyte antigen presentation, to inhibit the activation and proliferation of T lymphocytes, and to modulate the T cell-mediated cytokine response.

### The plasticity region cluster

The fascinating genomic landmark discovered post genomic era in both the sequenced strains is the one where 48% and 46% of the strain specific genes are located in J99 and 26695 respectively. This region is called as the 'plasticity zone' [28]. Genome sequence comparisons have revealed that nearly half of the strain specific genes fall in this zone. Recently, a new type IV secretion apparatus has been located in this plasticity zone [70]. This type IV cluster is comprised of 7 genes, homologous to the *vir *B operon of *A. tumifaciens *carried in a 16.3 kb genomic fragment called tfs3 (Figure 1). This cluster was discovered by Kersulyte *et al*. as a result of subtractive hybridization and chromosome walking and sequence homology. They also tested conservation of this island in clinical isolates and found that full length and partially disrupted tfs3 occur in 20% and19% of the strains respectively, from Spain, Peru, India and Japan. Although there is no correct role assigned to this cluster, it might be an unusual transposon linked to many deletion events occuring in the plasticity region that contribute to bacterial fitness in diverse host populations via exercising flexibility in gene content and gene order. The plastic nature of *H. pylori *and the evidence of horizontal transfer of genes from other *H. pylori *isolates and bacterial species could explain the ability of this organism to persist in a changing environment and why only a subset of clinical isolates exert an adverse effect on patients.

#### Link with cancer: are plasticity region genes involved?

The plasticity region as a whole displays certain characteristics of pathogenicity islands [71] with relatively low G+C content (35%) compared to the rest of the genome (39%). This region is about 45 kb long in J99 and 68 kb long in strain 26695. Genomic analysis revealed the region to be highly mosaic with a majority of the genes being transcribed suggesting their functional role. They also express protein level homology to various other recombinases, integrases and topoisomerases [72] accounting to natural transformation and recombination. In addition to these, many ORFs were identified as differentially expressed (JHP0927-JHP0928-JHP0931 and JHP-042-JHP0944-JHP0945-JHP0947-JHP0960). They share same chromosomal orientation and therefore they potentially represent a bacterial operon. It is interesting to study the expression or suppression of these ORFs in strains linked to different clinical conditions. Recent studies have posed a possibility to explore the presence of any new pathogenicity markers in the plasticity zone, although the functions of most of the putatively encoded proteins in this cluster are unknown. But they are thought to play a role in increasing the virulence capacity of *H. pylori *strains either directly or by encoding factors that could lead to variance in the clinical out come of the infection. More interestingly, it is also noted that some of the genes of the plasticity regions were co-inherited along with *cagA*. However their co-association with the disease status or with the severity of gastric inflammation was not established either due to small sample size or lack of clinical information [73]. Interestingly, a novel pathogenicity marker, JHP947 has been detected within the plasticity zone [74]. Many genes putatively linked to the development of gastric cancer have been assigned to the plasticity zone [72]. Researchers have looked for genetic markers in *H pylori *strains isolated from patients with gastric extranodal marginal zone B cell lymphoma (MZBL) of the mucosa-associated lymphoid tissue (MALT)-type and strains from age matched patients with gastritis only [75]. Two ORFs were significantly linked with gastric MZBL over gastritis strains: JHP950 (74% *v *49%) and JHP1462 (26% *v *3%). JHP950 proved specific for gastric MZBL when tested against a group of strains from patients with duodenal ulcer and patients with adenocarcinoma, with significant prevalence (49% and 39%, respectively), and is therefore the candidate marker for gastric MZBL. Interestingly, the candidate ORF JHP950 is located in the plasticity region of the J99 genome [75,76]. In view of such findings it can be speculated that some members of the plasticity region cluster provide selective advantage to some of the strains to adapt to changing host niches and become more and more invasive. In what way such advantage is gained? This needs to be discovered.

### Do we need to eradicate *H. pylori *from this earth?

How long humans carried *H. pylori *is still controversial. However, it is accepted that this organism has colonized humans possibly for many thousands of years, and the successful persistence of *H. pylori *in human stomach for such a long period may be because this organism offers some advantages to the host. Unfortunately, the *H. pylori *infection is on steep decline in the western world. This is mainly due to the success rate of combination therapies and subsequent prevention of re-infection due to improvement in sanitation and personal hygiene. This may seem good news to many gastro-enterologists around the world, but having a *H. pylori *infection may be advantageous. A study has shown that *H. pylori *produces a cecropin-like peptide (antibacterial peptide) with high antimicrobial properties [77]. A German study revealed that children infected with *H. pylori *were less likely to have diarrhoea than children without an infection [78], implying that *H. pylori *may have beneficial properties to human hosts. Interestingly, there has been a marked decline in the instances of peptic ulcer disease and gastric cancer in the 20^th ^century. Concurrent with this is a dramatic increase in the incidences of gastro-oesophageal reflux disease (GERD), Barrett's oesophagus and adenocarcinoma of the oesophagus in Western countries [79]. This observation led to the speculation that *H. pylori *may in some way be associated with these diseases and perhaps capable of preventing their onset. Studies have also shown that *cagA*^+ ^*H. pylori *strains have a more protective effect than *cagA*^- ^strains [80]. The presence of *cagA*^+ ^*H. pylori *strains can reduce the acidity of the stomach, and it is believed that the raising of the pH by *H. pylori *prevents GERD, Barrett's oesophagus and adenocarcinoma of the oesophagus (Figure). Conversely, arguments have been made that, although *H. pylori *may prevent these reflux-associated diseases, the risks of acquiring gastric cancer via *H. pylori *infection far outweigh any possible benefits it may provide [81]. However, it has been stated that, if *H. pylori *does provide protection from GERD, the notion of restriction of anti-*H. pylori *treatment to only a few cases (peptic ulcer disease and MALT lymphoma) could be justified [82]. In spite of this controversy, recent reports have demonstrated a protective role for *H. pylori *in erosive reflux oesophagitis [83-85]. However, as safe and potent anitsecretory drugs to prevent gastro-oesophageal reflux are available [86] it seems unjustified to use a dangerous organism that has been associated with extremely dangerous outcomes such as a carcinoma.

On the other hand, eradication also is not an ultimate choice. Some ulcers recur even after successful eradication of *H. pylori *in non-users of non-steroidal anti-inflammatory drug (NSAID). In addition, the incidence of *H. pylori*-negative, non-NSAID peptic ulcer disease (PUD) (idiopathic PUD) is reported to increase with time. Moreover, it appears that *H. pylori*-positive ulcers are not always *H. pylori*-induced ulcers because there are two paradoxes of the *H. pylori *myth, first the existence of *H. pylori*-positive non-recurring ulcer and secondly, recurring ulcer after cure of *H. pylori *infection. To summarise, *H. pylori *is not the only cause of peptic ulcer disease. Therefore, it is still necessary to seriously consider the need for eradication in all cases of PUD, which may exist even after the elimination of *H. pylori*.

### Conclusion and expert opinion

In our opinion, the intricacies of the role of *H. pylori *in health and disease may be fully ascertained only if we analyze genetic diversity of the pathogen as juxtaposed to the host diversity and the environment (food and dietary habits). A possible working hypothesis (that we are currently nurturing) may be that among the ocean of molecular host-pathogen interactions that could potentially occur with micro-evolution of this bacterium during long term colonization, some could prove advantageous where the bacterium and the host negotiate nearly a 'symbiotic' and balanced relationship. Such a 'friendship' might have taken thousands of years to develop. If so, why has this bacterium survived for such a long time? Microbes that have long been persisted in humans may be less harmful than recently emerged microbes, such as the human immunodeficiency viruses (HIV). This suggests that the colonization may either be beneficial or of low biological cost to the host. In addition to characterization of bacterial virulence apparatuses that are for sure linked to disease outcome, host responses to such factors must also be examined hand in hand, to completely ascertain mechanisms that lead to gastroduodenal disease. For instance polymorphisms linked to the host immune apparatus, such as *IL-1β*, *TNF-α*, and *IL-10*, which are responsible for elevated proinflammatory potential of the strains. These polymorphisms increase the risk for atrophic gastritis and distal gastric adenocarcinoma among *H. pylori*-infected persons. Cancer of stomach is a highly lethal disease and establishment of *H. pylori *as a risk factor for this malignancy deserves an approach to identify persons at increased risk; however, infection with this organism is extremely common and most colonized persons never develop cancer. Thus, screens to identify high-risk subpopulations must use high-resolution biological markers. Fortunately, this task appears to be highly simplified due to the availability of biological tools, which were never thought in the past. Genome sequences (*H. pylori*, human, *C. elegans*), quantitative phenotypes (cagA phosphorylation, *oip*A frame status, *vac *Aallele status), and practical animal models (Mongolian gerbils) can be harnessed to decipher the molecular basis of *H. pylori*-associated malignancies, which should have direct clinical applications. It is important to gain more insight into the pathogenesis of *H. pylori*-induced gastric adenocarcinoma, not only to develop more effective diagnostics and treatment for this common cancer, but also to validate the role of chronic inflammation in the genesis of other tumours of the alimentary tract.
